# Preemptive Analgesia in Total Knee Arthroplasty: Comparing the Effects of Single Dose Combining Celecoxib with Pregabalin and Repetition Dose Combining Celecoxib with Pregabalin: Double-Blind Controlled Clinical Trial

**DOI:** 10.1155/2018/3807217

**Published:** 2018-08-07

**Authors:** Andri M. T. Lubis, Rangga B. V. Rawung, Aida R. Tantri

**Affiliations:** ^1^Department of Orthopaedic and Traumatology, Cipto Mangunkusumo National Central Hospital and Faculty of Medicine, Universitas Indonesia, Jalan Diponegoro No. 71, Central Jakarta, Jakarta 10430, Indonesia; ^2^Department of Anesthesiology and Intensive Care, Cipto Mangunkusumo National Central Hospital and Faculty of Medicine, Universitas Indonesia, Jalan Diponegoro No. 71, Central Jakarta, Jakarta 10430, Indonesia

## Abstract

Acute pain is the most common early complication after total knee arthroplasty causing delayed mobilization and increased demands of morphine, leading to higher operative cost. Several studies have assessed the effectiveness, side-effects, and ease of use of various analgesics. Preemptive analgesia with combined celecoxib and pregabalin has been reported to yield positive outcomes. In this randomized, double-blind controlled clinical trial, 30 subjects underwent surgery for total knee arthroplasty using 15-20mg bupivacaine 5% epidural anesthesia. All subjects were divided into three groups. Group 1 was given celecoxib 400mg and pregabalin 150mg 1 hour before the operation, Group 2 was given celecoxib 200mg and pregabalin 75mg twice daily starting from 3 days before the operation, and Group 3 was given a placebo. The outcome was measured with Visual Analog Scale, knee range of motion, and postoperative mobilization. There was a significant difference in postoperative morphine usage between the groups that were administered with preemptive analgesia and the placebo group, but no significant difference was found between Group 1 and Group 2 that were given preemptive analgesia at different doses. ROM and postoperative mobilization were not significantly different among the three groups. Two patients in the first group, one patient in the second group, and one patient in the third group developed nausea. Preemptive analgesia is proven to reduce postoperative usage of morphine independent of the dosage. We recommend the use of combined celecoxib and pregabalin as preemptive analgesia after the total knee arthroplasty procedure. This trial is registered with NCT03523832 (ClinicalTrials.gov).

## 1. Introduction

Total knee arthroplasty (TKA) is one of the most common operation procedures in the orthopedic field [1]. Several studies have proved that the TKA procedure increases patient Quality of Life [2, 3]. Crowder et al. [4] reported that, after 10 years of follow-up, 99% of the patients that underwent the TKA procedure showed good functional outcome. The TKA procedure is indicated for patients experiencing chronic pain caused by severe joint damage. Although this procedure frequently yields positive clinical outcomes, complications during and after surgery can occur [5].

Acute pain following TKA is reported as one of the most common complications of the procedure [6, 7]. Several factors may contribute to the emerging pain sensation, such as infection, inflammatory response to the procedure, and race [8]. Effective postoperative pain management is crucial to promote recovery and to prevent complications. Although the mainstay of postoperative pain management has been opioid based, the consequent increase in demand of opioid use and the associated increase of opioid side-effects impede the rehabilitation process and increase the hospital length of stay, which in turn increases treatment cost and impacts patient satisfaction [8, 9].

The prevalent use of multimodal analgesia both intraoperatively and postoperatively has been described recently [10, 11]. Multimodal analgesia is a rational approach to pain management since no single analgesia targets all types of pain [12]. Epidurals, peripheral nerve blocks, and local articular injections have been reported to give good results; however they are associated with many side-effects such as headache, hypotension, neurologic bladder, and contralateral numbness [13, 14]. Patient Control Analgesia (PCA) using morphine is also reported to reduce postoperative acute pain, but it is associated with side-effects such as nausea and vomiting [15, 16].

Preemptive analgesia is intended to help cope with the pain sensation before the operative procedure. The aim of this therapy is to prevent central pain sensitization through the inhibition of pain stimuli and afferent signaling from the operation site [17–19]. A literature review by Katz and Clarke demonstrates the benefits of preemptive analgesia in various surgical procedures [20]. Decreasing pain sensation and lower postoperative analgesia usage was described in 60% of the cases in a study by Lee et al. [12]. Many preemptive analgesics can be taken postoperatively, such as nonsteroidal anti-inflammatory drugs (NSAIDs), N-methyl-d-aspartate (NMDAs), local anesthesia, and systemic antiepileptic drugs [12].

The use of oral preemptive analgesia is associated with positive outcomes [21]. Celecoxib (an oral NSAID) and pregabalin (an antiallodynic and antihyperalgesic) can be used as preemptive analgesia as their synergic effects may inhibit pain stimuli in different ways. Celecoxib has a better analgesic effect with lower rate of side effect compared to ibuprofen, and pregabalin has a similar analgesic effect with a lower rate of side-effects compared to gabapentin [22, 23]. A study by Niruthisard et al. showed that the combination of celecoxib and pregabalin taken 1 hour before the TKA procedure helps decrease pain during motion and postoperative anxiety [24]. Despite these promising effects, research on the repetitive dose of preemptive analgesia in the TKA procedure is limited. The aim of this study was to compare the therapeutic effect of single dose and repetitive dose of combined celecoxib and pregabalin.

## 2. Materials and Methods

All procedures undertaken in this study have been approved by The Ethical Committee of Faculty of Medicine Universitas Indonesia no. 851/UN2.F1/ETIK/2015. Verbal and written informed consent were obtained before the study. The study was carried out in accordance with the approved guidelines.

This study used a double-blind control clinical trial design conducted between July 2015 and December 2016. The sample size of this study was calculated based on formula of mean difference between unpaired numeric data in analytic research design. The sample size of 10 patients per group was adequate.

(1) n=2Ζα−Ζασμ1−μ22n=21.96−−0.841261602=9,7≃10  subjects

Patients were included if they met the following eligibility criteria: patients who (1) are aged between 55 and 80 years who visited the orthopedic polyclinic, (2) underwent the TKA procedure, (3) have osteoarthritis, and (4) consumed a pain killer and anti-inflammatory drug routinely. Patients who met all of the aforementioned criteria were consecutively enrolled into the study. Patients were excluded if they had (1) a psychiatric disorder, (2) history of renal disease (3) history of chronic neuropathy, having knee arthritis caused by rheumatoid arthritis and infection, (4) diabetes and obesity, (5) coagulopathy, and (6) severe pain that needed immediate analgesia.

The eligible subjects of the study were collected by consecutive sampling. The tested subjects were randomly allocated into three groups: Group 1: patients administered with a single dose of celecoxib 400 mg and pregabalin 150 mg, Group 2: patients administered with a repetitive dose of celecoxib 200 mg and pregabalin 75 mg twice daily for 3 days before the operation, and Group 3: patients taking a placebo. This study used 3 groups to compare repetitive dose of oral preemptive analgesia with the more common preemptive analgesia treatment of single dose combining celecoxib with pregabalin and control group. Currently no research had been done to analyze the effect of repetitive dose of preemptive analgesia in TKA procedure according to mechanism of administration, dosage, and efficacy.

This study used block randomization with the size of 3 according to each group. Block randomization was used to reduce bias and achieve balance in the allocation of participants to treatment arms, especially when the sample size is small (10 samples). This method increases the probability that each arm will contain an equal number of individuals by sequencing participant assignments by block. Allocation proceeds by randomly selecting one of the orderings and assigning the next block of participants to study groups according to the specified sequence. The double-blind design in this study included blinding of orthopedic surgeon, anesthetic operator, medical team giving the drugs, and the patients.

All surgeries were performed by an experienced orthopedic surgeon. All groups underwent the same procedure for epidural anesthesia with 15-20 mg bupivacaine 5% before the TKA procedure. Two hours after the procedure, postoperative analgesia, which consisted of paracetamol 1000 mg + PCA morphine 2 mg/kgBW with maximal dose of 6 mg/hr and 5 minutes' interval, was administered.

The efficacy of preemptive analgesia among the groups was measured during the postoperative follow-up using the outcome parameters listed below. All side-effects of postoperative therapy were monitored and treated.

### 2.1. Outcome Parameters

#### 2.1.1. Total Morphine Consumption

This was the primary outcome parameter to measure the efficacy of the preemptive analgesia of combined celecoxib and pregabalin. In this study, morphine was administered by patient control analgesia (PCA). Total morphine consumption was measured postoperatively for 3 days.

#### 2.1.2. Postoperative Pain

The acute pain is measured and evaluated by Visual Analog Scale (VAS) every morning before the patients began their rehabilitation program. This parameter is measured from the first day until the third postoperative day (0-24 hours, 25-48 hours, and 49-72 hours).

#### 2.1.3. Knee Functional Outcome

The knee range of motion was measured in the morning from the first day until the third postoperative day. The patient was asked to actively move the knee joint, and the knee angle was measured by goniometer.

#### 2.1.4. Mobilization

The patient was expected to be able to sit on the first day, stand on the second day, and walk on the third day in the postoperative period.

### 2.2. Statistical Analysis

The outcomes of this research were numerically expressed and the differences among the three groups were measured within the first day until the third postoperative day.

Statistical analysis was performed with SPSS software version 20 (SPSS Inc., Chicago, IL, USA). The normality of distribution for numeric variables was assessed by the Shapiro-Wilk test. Accordingly, categorical variables are presented as means with SD or medians with interquartile range (95% CI). Data were tested using a one-way ANOVA if the data were normally distributed and with the Kruskal-Wallis test if they were not normally distributed. Any significant difference found by the one-way ANOVA was then analyzed by the Bonferroni post hoc test to assess the significance of each group.

## 3. Results and Discussion

A total of thirty subjects were included in this study. Each group consisted of ten randomized subjects. The characteristics of the patients are described in Table 1.

### 3.1. Total Morphine Consumption

There was a significant difference in the total morphine consumption among the 3 groups (p<0.001). The results of morphine consumption measurement are summarized in Table 2.

The post hoc Bonferroni test concluded a significant difference in the total morphine consumed between the first and third group (p<0.001), as well as between the second and the third group (p<0.001), but there were no significant differences between the first and the second group.

### 3.2. Postoperative Pain

Postoperative pain was measured with VAS. The measurement was done on the first day until the third day. The VAS within each group generally decreased from Day 1 until Day 3. Significant differences in VAS scores between the three groups were observed (p<0.001) on the first day, second day, and the third day. The third group showed higher score of VAS than the first and the second group. The VAS measurement among the groups evaluated from the first until third day can be seen on Table 3.

The post hoc Bonferroni test concluded a significant difference of total morphine consumption between the first and third group (p<0.001) and between the second and the third group (p<0.001), but there were no significant differences between the first and the second group.

### 3.3. Knee Functional Outcome

The range of motion in all groups improved from Day 1 until Day 3. There was no significant difference in ROM among all groups on the first day (p=0.886), on the second day (p=0.131), and on the third day (p=0.011). Nevertheless, the clinical outcome of knee measurement using goniometer showed daily improvement in ROM. Knee functional outcome measurement among the groups after undergoing TKA procedure can be seen on Table 4.

### 3.4. Mobilization

Almost all of the subjects from each group were able to sit on the first day, stand on the second day, and walk on the third day. There was no significant difference among the groups (p=1.00). Mobilization measurement among the groups after undergoing TKA procedure can be seen on Table 5.

Osteoarthritis often affects geriatric patients. Its incidence is impacted by age as well as gender, wherein females are more affected than men [25]. These demographic characteristics were also found in our patient sample. As more than half of the patients undergoing TKA are either late adult or geriatric, higher rates of complications can be expected. It follows that intensive evaluation is often required when using analgesia [26, 27]. In our study, the use of morphine was significantly lower in the groups that received preemptive analgesia regardless of the duration compared to the placebo group. Our findings are in line with a previous study conducted by Lee et al. [28] and Huang Yu et al. [29] which found that the use of celecoxib (400 mg) combined with pregabalin (150 mg) given 1 hour prior to the TKA procedure lowered the use of morphine and postoperative pain. However, our study found no significant difference in the time of administration of the preemptive analgesia combination (1 hour or 72 hours) prior to the procedure.

In terms of efficacy, we found that VAS in the groups that were taking preemptive analgesia was lower than the placebo group. Similar results have been described by Buvanendran et al. [19] and Carmichael et al. [30] who found that the use of repetitive doses of celecoxib and pregabalin succeeded in lowering acute and chronic postoperative pain. Although we did not measure the outcome of chronic pain among the treatment groups, it was found that, in general, the VAS score decreased from Day 1 to Day 3 and significantly differed from the group that used either a single dose or a repetitive dose of preemptive analgesia compared to the placebo group. Other confounding factors such as race, gender, and the method of VAS measurement warrant further consideration in future studies.

The rehabilitation program is an important aspect of postoperative recovery. It has been reported that early mobilization following the TKA procedure results in a better outcome such as lower morbidity and hospital length of stay [31]. It follows that ROM assessment is important to ensure early mobilization of patients. The fact that ROM is directly affected by pain implies that lowering postoperative pain with preemptive analgesia will increase ROM and early mobilization rate of the patients. In this study, ROM was found to improve day to day, but no significant difference was observed in ROM between subjects that were given preemptive analgesia compared to placebo group. According to Miner et al. [31] and Jacobsen et al. [32], the use of ROM as the outcome parameter after the TKA procedure is still controversial. Their studies report that many factors like knee soft tissue, knee alignment, knee muscle strength, psychological condition, preoperative ROM, and operative procedure impact the postoperative ROM. The result of our study supports the fact that ROM is not only affected by pain but also affected by multifactorial aspects that have not been studied in our research.

Preemptive analgesia is expected to permit the early mobilization of postoperative patients. Early mobilization reduces complications like DVT which can be avoided and promotes the healing process, which in turn improves functional outcomes [33, 34]. In this study, we did not find any significant differences in the mobilization period among the 3 groups. Bade reported that preoperative mobilization directly affected postoperative mobilization outcome. Many factors may affect postoperative mobilization, such as age, comorbidities, muscle strength, and psychological factors. The result of our study supported the fact that patient mobility is not only affected by postoperative pain and suggests the possibility that preemptive analgesia only lowers static postoperative pain, not lowering the pain sensation during mobilization.

Our study found that the side-effects were dose dependent and often emerged in patients using the celecoxib combination with pregabalin for prolonged periods. Buvanendran [19] reported that patients administered with 300 mg pregabalin 1 hour postoperatively experienced sedation, confusion, and dry mouth side-effects. Lee et al. [28] compared the use of the 400mg celecoxib and 150 mg pregabalin combination with 400mg celecoxib alone and found that the side-effects emerged in the combination group. In our study, we found that 2 subjects in first group, 1 subject in second group, and 1 subject in third group experienced nausea.

## 4. Conclusions

Preemptive analgesia treatments with the combination of celecoxib and pregabalin are effective in decreasing the acute pain sensation after the TKA procedure independent of the dose and repetition. This combination of preemptive analgesia is ineffective in decreasing pain sensation during motion. In this study, the use of combination of celecoxib and pregabalin did not show any significant side-effects.

## Figures and Tables

**Table 1:** The characteristics of the patients.

No	Variable	Group 1 (N=10)	Group 2 (N=10)	Group 3 (N=10)
1	Gender	Man (3) Woman (7)	Man (3) Woman (7)	Man (1) Woman (9)
2	Age (years)	66.1 ± 7.79	65.9 ± 5.82	68.2 ± 6.35

**Table 2:** Total morphine consumption (PCA) among the groups after undergoing TKA procedure.

	Group 1 (1 hr preemptive analgesia)	Group 2 (72 hrs preemptive analgesia)	Group 3 (Placebo)	P value*∗*
	N=10	N=10	N=10	
Morphine (PCA)	10.60(2.675)	9.90(1.524)	30.20(5.308)	<0.001

The data were normally distributed.

The p value was measured using ANOVA parametric test.

**Table 3:** VAS measurement among the groups after undergoing TKA procedure evaluated from the first day until the third day.

	VAS D-1	VAS D-2	VAS D-3	P value*∗*
	N=10	N=10	N=10	
Group 1 (1 hr preemptive analgesia)	2 (2-3)	1 (1-2)	1 (1-2)	<0.001
Group 2 (72 hrs preemptive analgesia)	2.5 (2-3)	2 (1-2)	1.2 (1-2)	0.001
Group 3 (Placebo)	4(2-5)	3 (2-4)	3 (2-3)	0.001

The data were not normally distributed.

The p value was measured using Kruskal-wallis nonparametric test.

**Table 4:** Knee functional outcome measurement among the groups after undergoing TKA procedure evaluated from the first day until the third day.

	ROM D-1	ROM D-2	ROM D-3
	N=10	N=10	N=10
Group 1 (1 hr preemptive analgesia)	15 (10-30)	60 (15-60)	90 (60-90)
Group 2 (72 hrs preemptive analgesia)	15 (15-20)	30 (30-60)	60 (45-90)
Group 3 (Placebo)	15(15-20)	30 (30-60)	60 (60-90)
p-value	0.886	0.131	0.011

The data were not normally distributed.

The p value was measured using Kruskal-wallis nonparametric test.

**Table 5:** Mobilization measurement among the groups after undergoing TKA procedure evaluated from the first day until the third day.

	Motion D-1 (sitting)	Motion D-2 (standing)	Motion D-3 (walking)	p value*∗*
	N=10	N=10	N=10	
Group 1 (1 hr preemptive analgesia)	9(90%)	8 (80 %)	10 (100%)	<0.001
Group 2 (72 hrs preemptive analgesia)	8 (80%)	7 (70 %)	7 (70%)	0.001
Group 3 (Placebo)	9 (90%)	7 (70%)	8 (80%)	0.001

The data were not normally distributed

The p value was measured using Kruskal-wallis non-parametric test.

## Data Availability

The data underlying the findings of the study could be obtained if a request is sent to the corresponding author's email: rangga_orthoui@yahoo.com
